# Genetics of hip dysplasia – a systematic literature review

**DOI:** 10.1186/s12891-024-07795-2

**Published:** 2024-10-01

**Authors:** Kaya Kvarme Jacobsen, Lene Bjerke Laborie, Hege Kristiansen, Annette Schäfer, Trude Gundersen, Tetyana Zayats, Karen Rosendahl

**Affiliations:** 1 Department of Clinical Medicine, University of Bergen, Bergen, Norway; 2 Department of Orthopedic Surgery, District General Hospital of Førde, Førde, Norway; 3 Section for pediatric radiology, Department of Radiology, Haukeland University Hospital, Bergen, Norway; 4 Department of Paediatrics, District General Hospital of Førde, Førde, Norway; 5 Department of Clinical Science, University of Bergen, Bergen, Norway; 6 Department of Orthopaedic Surgery, Haukeland University Hospital, Bergen, Norway; 7 Analytic and Translational Genetics Unit, Massachusetts General Hospital, Boston, MA USA; 8 Stanley Center for Psychiatric Research, Broad Institute of MIT and Harvard, Cambridge, MA USA; 9 Department of Psychology, PROMENTA, University of Oslo, Oslo, Norway; 10 Department of Radiology, University Hospital of North-Norway, Tromsø, Norway; 11 Department of Clinical Medicine, UiT, The Arctic University of Norway, Tromsø, Norway

**Keywords:** Developmental dysplasia of the hip, DDH, Genetics, GDF5, Heritability

## Abstract

**Background:**

Developmental dysplasia of the hip (DDH) is a congenital condition affecting 2–3% of all newborns. DDH increases the risk of osteoarthritis and is the cause of 30% of all total hip arthroplasties in adults < 40 years of age. We aim to explore the genetic background of DDH in order to improve diagnosis and personalize treatment.

**Methods:**

We conducted a structured literature review using PRISMA guidelines searching the Medline, Embase and Cochrane databases. We included 31 case control studies examining single nucleotide polymorphisms (SNPs) in non-syndromic DDH.

**Results:**

A total of 73 papers were included for full text review, of which 31 were single nucleotide polymorphism (SNP) case/control association studies. The literature review revealed that the majority of published papers on the genetics of DDH were mostly underpowered for detection of any significant association. One large genome wide association study has been published (*N* = 9,915), establishing *GDF5* as a plausible risk factor.

**Conclusions:**

DDH is known to be congenital and heritable, with family occurrence of DDH already included as a risk factor in most screening programs. Despite this, high quality genetic research is scarce and no genetic risk factors have been soundly established, prompting the need for more research.

**Supplementary Information:**

The online version contains supplementary material available at 10.1186/s12891-024-07795-2.

## Introduction

Developmental dysplasia of the hip (DDH) is a congenital hip disorder characterized by a shallow or dysplastic acetabulum, with or without a dislocatable or dislocated femoral head. Based on ultrasound diagnosis, DDH is found in 2–3% of newborn children and is more common in females (5.7%) than in males (1.2%) [1, 2]. A Norwegian cohort study of healthy individuals at skeletal maturity (18–19 years of age) found a prevalence of DDH between 1.7 and 20%, depending on the type of radiological measurement used. As many as 3.1% of participants had two or more criteria for acetabular dysplasia [3].

DDH leads to pain, gait issues and increased risk of osteoarthritis (OA), and affects quality of life [4, 5]. The introduction of systematic ultrasound diagnostics including dynamic examination has increased the number of patients identified early enough to recieve low risk, non-invasive abduction device treatment rather than procedures such as hip reduction, orthosis treatment or osteotomies of the pelvis and/or femur [6]. In Norway, presence of clinical risk factors, such as familiar occurrence and breech delivery, and/or positive Barlow and/or Ortolani clinical tests at birth, warrants referral to the selective ultrasonographic screening in the newborn period [7]. Even with several known risk factors, the introduction of ultrasound screening of newborn children with risk factors for DDH has not significantly impacted the incidence of late diagnosis of DDH, although some reduction has been shown [6, 8]. These findings highlight the need for improved diagnosis of DDH based on increased knowledge of the disorder.

The congenital aspect of DDH has been proven for over 50 years. Czeizel et al. found a reduced Wiberg angle in the parents of children with DDH in 1975 [9]. This has been further supported through a RR for DDH in first degree relatives as high as 12.1 [10]. Through twin studies the proportion of DDH phenotypic variance due to genetic factors has been calculated to be approximately 74% in Norway and 84% in Han Chinese individuals [11, 12]. This establishes a substantial genetic contribution to DDH and suggests a difference in risk factors and possibly in genetic background for both DDH and OA in different ethnic groups.

Despite the known familial component, no genetic test exists to determine the risk of DDH and very little is known about the genetic pathophysiology of DDH. In this review, we aim to summarize recent progress within the genetic field of DDH. We focus on the studies on single nucleotide polymorphisms (SNPs) in DDH, but also give a brief summary of SNP studies of primary osteoarthritis in order to compare this to development of osteoarthritis secondary to DDH.

## Methods

**Fig. 1:**
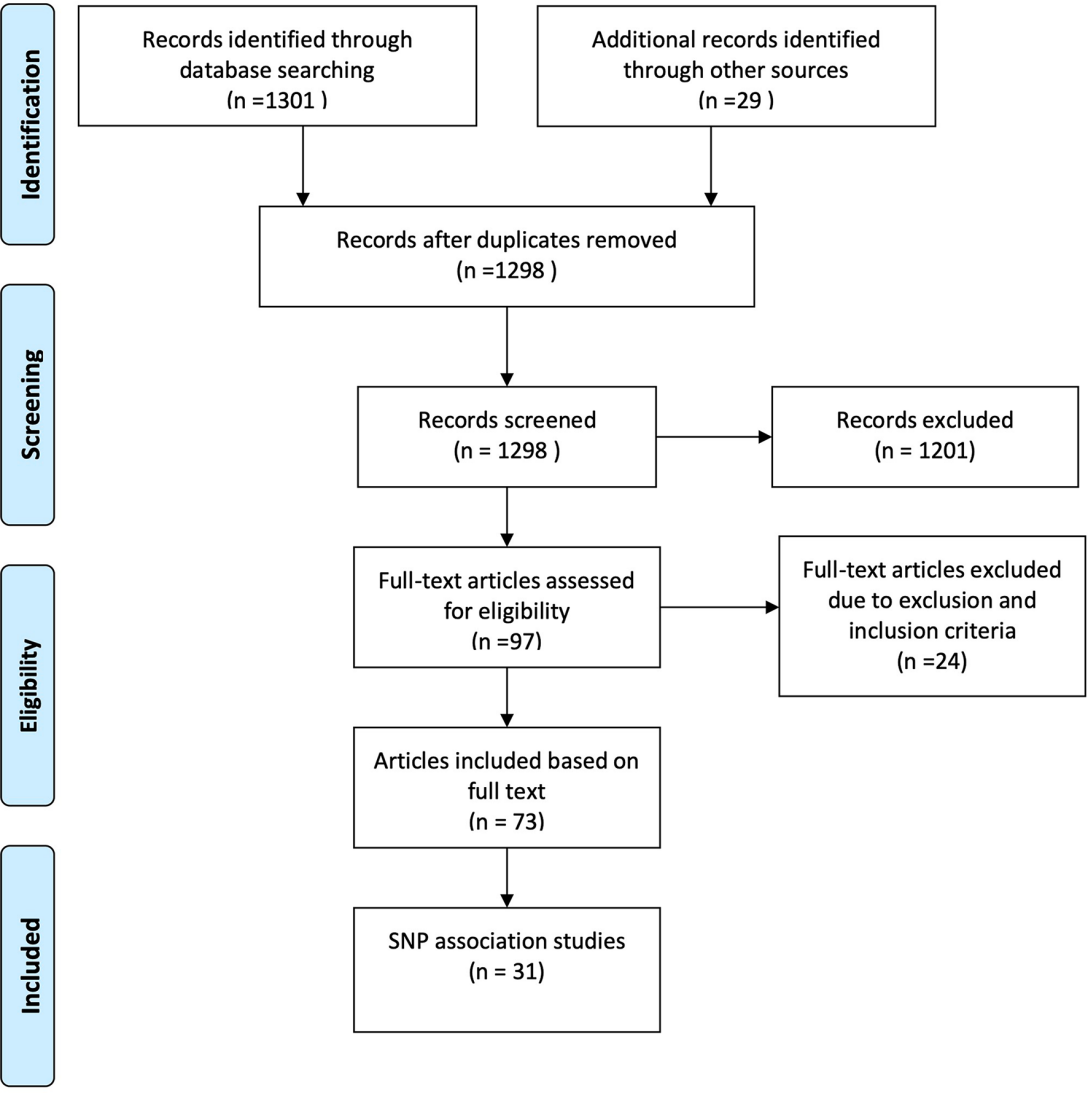
PRISMA diagram of the review workflow

This systematic literature review was performed in accordance with the PRISMA guidelines [13]. We searched Medline, Embase and Cochrane databases on 6th of February 2023, using search terms «Hip dysplasia», «congenital dislocation of the hip» or «congenital hip dysplasia» in combination with «Genetic» or «Gene». A detailed list of search criteria and terms is available in Appendix A. The search terms were chosen in order to include studies using both older and more current terms of hip dysplasia as well as a wide spectrum of genetic studies. Studies on canine hip dysplasia, syndromes where hip dysplasia is a minor feature, different topics erroneously included by the wide inclusion criteria as well as studies without genetic focus (e.g. clinical treatment case reports) or written in languages other than English were excluded. We did not set a date limit in the search, but as we focused on the modern method of SNP-based case/control analyses, the results were limited to the era of such studies. All abstracts not affected by the exclusion criteria were reviewed and a study was included if it met the inclusion criteria above and its full text article was available either through library subscriptions or direct contact with the author(s), otherwise only the abstract was evaluated (and noted in the corresponding text). Figure 1 depicts a detailed overview of the process.

Screening and selection of the search results were performed using the Rayyan software, with authors KKJ and LBL including/excluding studies [14]. When reviewing the included papers, we focused on the number of cases and controls included, the percentage of women in both groups, the ethnicity of the included individuals, method of defining cases and selecting controls, association with tested SNPs and control of multiple testing. We chose these parameters in order to evaluate the statistical power as well as strengths and weaknesses of each study.

## Results

The systematic literature search identified a total of 73 studies after full text review. Forty-two were a mixture of family-based genetic studies, studies of other types of genetic variants and functional studies, while 31 were case control SNP association studies. Of these, 18 were studies in Han Chinese populations, three in Middle Eastern populations and ten in European populations. Twentynine of the SNP studies were candidate gene studies and only three studies were genome-wide association studies.

Table 1 and Supplementary Table 1 summarize the details of the 31 papers, with Table 1 focusing on clinical data and Supplementary Table 1 on statistical analyses and results. In several of the studies, the power was too low to detect significant association, an issue that was frequent in other disorders using genetic association studies before larger samples and collaborations were established.

**Table 1:** Summary of the 31 SNP-based studies included

Paper	Ethnicity	Type of study	Diagnosis of DDH	Measure-ment	No Cases	No Controls	Gender difference	Gender control	Multiple testing correction
Cengic et al., Int Orthopedics 2015^10^	Slavic	Candidate gene	X-ray	CEA	68	152	Yes	Yes	No
Dai et al., Arthritis Res Ther. 2008^16^	Han Chinese	Candidate gene	Radiology	No info	338	622	Yes	Subgroup analysis	No
Ghosh et al., Med Sci Monit^20^	Western European	Candidate gene	Radiology	AI and CEA	45	95	Yes	No	No
Gumus et al., Indian J Orthop 2021^21^	Turkish	Candidate gene	Radiology	No info	68	100	Yes	No	No
Hao et al., J Orthop Res. 2014^22^	Han Chinese	Candidate gene	Radiology	No info	460	562	Yes	Subgroup analysis	No
Harsanyi et al., Ortop Trauma Rehab 2021a^23^	Slavic	Candidate gene	Ultrasonography	Graf	35	83	Yes	No	No
Harsanyi et al., Ortop Trauma Rehab 2021b^24^	Slavic	Candidate gene	Ultrasonography	Graf	45	85	Yes	No	No
Hatzikotoulas et al., Commun Biol. 2018^25^	Western European	GWAS	X-ray in subsample	No info	1899	8016	Yes	Yes	Yes
Igrek et al., Acta Chir Orthop Traumatol Chec 2021^27^	Slavic	Candidate gene	Radiology	No info	105	119	Yes	Subgroup analysis	No
Jawadi et al., J Genet. 2018^28^	Arabic	Candidate gene	No info	No info	50	50	Yes	No	No
Jia et al., Osteoarthritis Cartilage. 2012^29^	Han Chinese	Candidate gene	Radiology	No info	310	487	Yes	Yes	No
Kapoor et al., J Negat Results Biomed. 2007^30^	Western European	Candidate gene	X-ray	AI and CEA	45	101	Yes	No?	Yes
Kolundžić et al., Cytokine. 2011^33^	Slavic	Candidate gene	Radiology	No info	28	20	Yes	Yes	No
Li et al., J Orthop Res. 2017^36^	Han Chinese	Candidate gene	Radiology	No info	689	689	Yes	Yes	No
Ma et al., Sci Rep. 2017^37^	Han chinese	Candidate gene	Radiology	No info	691	2027	No	Yes	Yes
Qiao et al., Int J Exp Pathol 2017a^44^	Han Chinese	Candidate gene	Radiology	Complete dislocation	409	351	?	Subgroup analysis	No
Qiao et al., Int J Exp Pathol 2017b^45^	Han Chinese	Candidate gene	Radiology	Complete dislocation	984	2043	?	Subgroup analysis	No
Rouault et al., Osteoarthritis Cartilage 2009^51^	French	Candidate gene	Radiology	Graf/Wilson, CEA, HTE	239	239	No	Yes	Yes
Rouault et al., Osteoarthritis Cartilage 2010^52^	French	Candidate gene	Radiology	Graf/Wilson, CEA, HTE	239	239	No	Yes	Yes
Sadat-Ali et al., Biocehm genet 2018^53^	Arabic	Candidate gene	Radiology	No info	100	100	?	No	No
Shi et al., BioMed Res Int 2014^55^	Han Chinese	Candidate gene	Radiology	No info	697	707	Yes	Subgroup analysis	No
Sun et al., PLoS ONE 2015^59^	Han Chinese	GWAS	Radiology	No info	755	944	?	No	No
Sun et al., Sci Rep 2019^60^	Han Chinese	Candidate gene	Radiology	No info	200	429	?	No	No
Tian et al., BMC Musculoskelet Disord 2012^65^	Han Chinese	Candidate gene	Radiology	No info	209	173	No	Yes	No
Wang K et al., Osteoarthritis Cartilage 2010^66^	Han Chinese	Candidate gene	Radiology	No info	505	551	?	Yes	No
Xu et al., Int J Exp Pathol 2016^69^	Han Chinese	Candidate gene	Radiology	Complete dislocation	170	454	Yes	Subgroup analysis	No
Xu et al., Aging, 2020^68^	Han Chinese	Candidate gene	Radiology	No info	350	595	Yes	Subgroup analysis	No
Yan et al., Clinical Genet 2019^70^	Han Chinese	GWAS	X-ray	AI	960	1069	?	Yes	Partially
Zhang et al., Gene 2018^71^	Han Chinese	Candidate gene	Radiology	No info	386	558	?	No	No
Zhao et al., Sci China Life Sci 2013^72^	Han Chinese	Candidate gene	Radiology	AI, CEA, AHI, Shenton	192	191	No	Yes	No
Zhu et al., Rheumatol Int 2011^73^	Han Chinese	Candidate gene	Radiology	No info	368	413	Yes	Subgroup analysis	No

CEA Center Edge Angle, AI: Acetabular Index, HTE: Horizontal Toit Extern angle, AHI: Acetabular head index, GWAS: Genome Wide Association Study, SNP: Single Nucleotide Polymorphism. “Radiology” implies that the modality was not further specified

Many of the authors did not correct for gender biases or multiple testing when interpreting the results. In addition, some studies seemed to overlap in sample origin without stating this clearly, most notably between genome-wide association studies (GWASs) and candidate gene studies. Most studies lack details on the clinical diagnosis and radiological measurements used to classify cases, making it difficult to compare the results across various studies. Overall, there is evidence for robust association signal in *GDF5* from both candidate gene studies and a GWAS [15–19]. Additional genes with some support for association include HOX-genes. In the following paragraphs a few highlights among the 31 papers are presented.

### SNP based candidate gene studies

Dai et al. looked at the rs143383 SNP in *GDF5* gene and found an association in a Han Chinese population [15]. The patients in the study consisted mostly of girls with dislocated hips (86%), while the control group was 51% females. Thus, it is likely that the association was primarily driven by sex, as sex was not considered in the reported analyses (OR 1.46 [95% CI: 1.21–1.91], *P* = 0.0053) [15]. Harsanyi et al. reported association with rs143383 in a Slavic population (OR not available, *P* = 0.047) [20]. Sadat-Ali et al. analyzed rs143383 in a Saudi population and did not find association in the case/control part of the study (*P* = 0.08). However, evidence suggested a paternal effect, as the T allele was more common in fathers and in affected children than in mothers [18]. Rouault et al. were able to confirm the association between *GDF5* and DDH through another SNP rs143384 (OR 1.53 [95% CI: 1.18–1.98], *P* = 0.002) in a French population [17]. Zhao et al. looked at a different Han Chinese population, analyzing two additional SNPs in *GDF5*, rs224332 and rs224333 and found association with DDH (OR not available, *P* = 0.001 and 0.006 respectively) [19]. *GDF5* is important for hip morphology and cartilage development [21].

Jia et al. found association between DDH and rs726252 in the *PAPPA2* gene in a Han Chinese sample (OR 1.83 [95%CI: 1.33–2.52], *P* = 0.001). The association was stronger in males (OR 3.69 [95% CI: 1.45–9.38], *P* = 0.006) than in females (OR = 1.605 [95% CI: 1.13–2.28], *P* = 0.008), although the number of affected males in the study was low (*N* = 57, 18% of cases) [22]. However, this finding was not supported in the larger study by Shi et al. in a separate Han Chinese sample (OR 0.89 [95%CI: 0.689–1.14], *P* = 0.36, *N* = 1404) [23]. Moreover, neither study corrected for multiple testing. *PAPPA2* regulates the function of growth factors, including insulin-like growth factor (IGF) [22]. In vitro and mice studies suggest that *PAPPA2* affects cartilage proliferation through regulation of the IGF-system, with a rat model of DDH showing altered IGF1-expression in the dysplastic hip [24, 25].

An association with rs1800470 in *TGFB1* was found in a one of the larger candidate gene studies included in this review (*N* = 4206) [26]. This was a study of Han Chinese by Ma et al., where most participants were women. The female ratio was 79% in both cases and controls in the discovery sample and 84% in the replication sample. The study found an association between *TGFB1* and a dislocated hip (OR 1.37 [95% CI: 1.12–1.68], *P* = 0.002) [26]. Contrary to many other papers, Ma et al. did report results corrected for multiple testing. *TGFB1* is a growth factor involved in bone remodeling and cartilage development [27].

Qiao et al. did a combined case/control study and family-based candidate gene study of *TXNDC3*, a gene involved in chondrocyte development and bone mineral density [28]. The case/control study included a discovery and replication stage, with a total of 3018 individuals of Han Chinese origin. The family-based part of the study included seven families, consisting of in total 15 non-affected individuals and 15 cases. In the total sample, a SNP in *TXNDC3* was significantly associated with DDH (OR 0,79, [95%CI: 0.62–0.93], *P* = 1,53 × 10^− 5^). The proposed protective allele had a lower frequency among the 15 DDH cases in the seven families compared to the 15 healthy family members, although no formal statistics was reported [28].

### DDH in the GWAS era

To date, the most comprehensive GWAS of DDH was published by Hatzikotoulas et al. in 2018 [16]. The authors reported a genome-wide significant association in the discovery sample with *GDF5* at rs143384 (OR 1.57 [95% CI: 1.3–1.77], *P* = 1.72 × 10 ^− 14^). The association was replicated in the larger replication sample (OR 1.37 [95% CI: 1.24–1.51], *P* = 1.33 × 10^− 10^). Meta-analysis further strengthened this finding (OR 1.44 [95% CI: 1.34–1.56], *P* = 3.55 × 10^− 22^). Additionally, the total SNP-based heritability of DDH on the liability scale in the discovery sample was calculated to be 55%, of which 0.96% points of the phenotypic variance were explained by the SNP that reached genome-wide significance in the meta-analysis. This suggests a large contribution to the phenotypic variance coming from genetic variants that did not reach genome-wide significance in this study and remain undetected. Gene-based analyses found five genes to be significantly associated with DDH: *GDF5*, *UQCC1*, *MMP24*, *RETSAT* and *PDRG1* (*P* = 9.24 × 10^− 12^; *P* = 1.86 × 10^− 10^; *P* = 3.18 × 10^− 9^, *P* = 3.7 × 10^− 8^, *P* = 1.06 × 10^− 7^) [16]. These genes are known to be involved in chondrogenesis, limb and joint development and extracellular matrix/collagen production. Surprisingly, polygenic risk score analyses and LD score regression did not find any genetic overlap between DDH and primary osteoarthritis in the UK biobank dataset [16].

Apart from clinical diagnosis, the DDH phenotype can also be examined as a femoroacetabular shape phenotype that may be seen as a continuum where the most extreme measurements include the classic pathological hip dysplasia shapes. A GWAS study on hip shape identified genome-wide associations with three different principal components of hip shape [29]. Genes implicated through this study included *ASTN2* and *PTHLH*, which have been associated with a greater risk of secondary OA in the arcOGEN study as well as the arOGEN/UKBB meta-analysis [29–31]. Several of the SNPs identified as genome-wide significantly associated with hip shape are located in genes known to be involved in endochondral bone formation, including *FGFR4*,* SOX9*,* HHIP*,* NKX3- 2*,* DICER1*, and *RUNX1* [29].

**Fig. 2:**
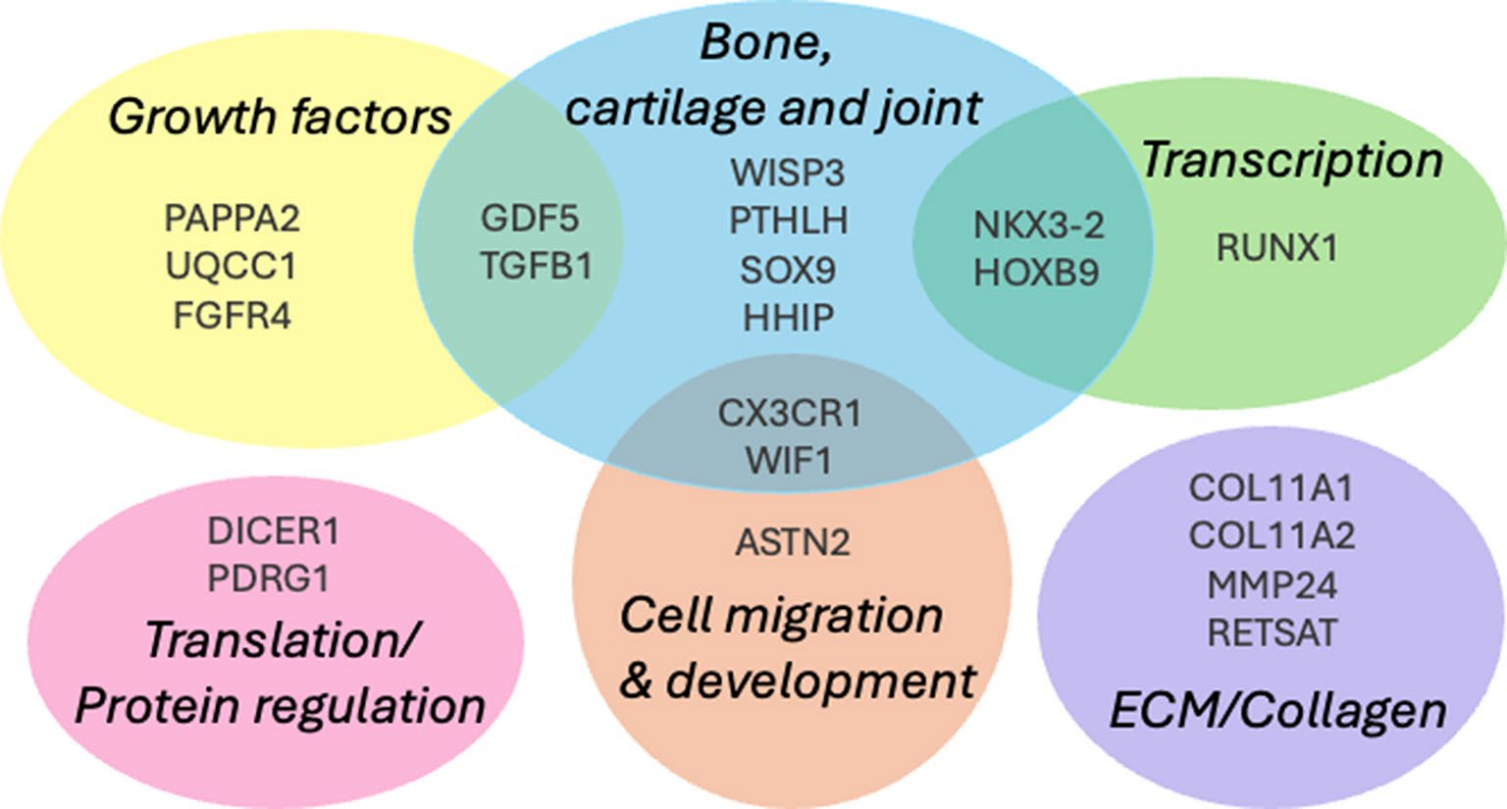
Genes found associated with DDH, grouped by function. Several are regulatory genes such as transcription and growth factors, and some are involved in bone, cartilage and joint development. ECM; Extracellular matrix

Figure 2 groups the genes from the studies reviewed that have been most strongly associated with DDH by function. Subgroups include bone, cartilage and joint development, regulation of transcription, translation and protein function including growth factors, cell migration/development and extracellular matrix development.

### Genetic studies of primary osteoarthritis

Although primary osteoarthritis is a multifactorial disorder, one can postulate that since DDH is a major risk factor for secondary osteoarthritis, there can either be a genetic overlap between the two, or some of the findings from genetic studies on primary OA can be driven by subpopulations where undiagnosed DDH results in a secondary OA. This is especially relevant in Japanese populations were DDH is the major cause of overall OA [32]. Even if any recognized secondary OA due to DDH is removed from the sample, a view of DDH as part of a continuous distribution of femoroacetabular shape leads to the theory that many primary OA cases are the result of unrecognized mild DDH. As such, it is relevant to briefly review the genetics of primary hip OA in the context of DDH.

Compared to DHH, the genetics of primary OA already have several established genome-wide associations [33][. Some of the larger GWA studies on primary hip OA exclude individuals with a diagnosis of DDH. However, the radiological basis for the diagnosis of DDH is rarely reported, creating uncertainty about the validity of the exclusion criteria [31, 34]. A patient-reported diagnosis of DDH has been shown in several studies to be quite accurate, but there could possibly be patients with OA secondary to an undiagnosed DDH misdiagnosed as idiopathic/primary OA. This will increase the heterogeneity in the case group of case/control studies on primary OA.

Genes associated with primary hip OA across at least two large GWASes (*N* > 100,000) include *IL11*,* COL11A1*, *GSDMC*,* TNC*,* LTBP3*,* HFE/HIST1H2BC*,* LMX1B* and *NACA2*. Pathway analyses linked these OA candidate genes to skeletal development or rare monogenic bone disorders [33]. *GDF5* was found to be associated with primary OA through early candidate gene studies, with alleles in the promotor region increasing the risk of OA possibly by decreasing the transcription of the gene [35].

### Summary

DDH is a musculoskeletal disorder which affects quality of life, often during the entire life span, even with state-of-the-art treatment. Although DDH is primarly considered a pediatric orthopedic condition, it does in fact influence both adolescence and adulthood. Thus, recognizing DDH as a lifelong disorder, contributing greatly to the number of young individuals with osteoarthritis and subsequent need of total hip arthroplasty (THA), is important. In addition to the impact on patient’s quality of life, the disorder also represents a significant cost to society. THAs in DDH patients are more expensive and time consuming than in primary OA patients and cost several thousand of euros [36–38]. One study found that the majority of DDH patients receiving THA had not been treated for DDH in childhood, supporting the notion that a substantial number of individuals do not receive a timely DDH diagnosis [36]. In summary, improvement in the diagnosis, follow up and treatment of DDH can greatly reduce future costs for patients and society, and an increased knowledge of genetics and the etiology of DDH will aid this effort.

The development of DDH is hypothesized to be due to a combination of genetic risk and mechanical stimuli, such as breech position, with complex epigenetic mechanisms and gene-environment interactions coming into play [39]. Hogervorst et al. postulated that different genetic variants determine both skeletal morphology (morphotype) as well as cartilage development and composition (cartilotype). As such, an individual can have a morphotype that increases the risk of OA (for instance a mild hip dysplasia), but a cartilotype with high ability to withstand the less favorable morphology. Thus, the individual never develops a clinically significant OA. In addition, fetal breech position could both alter gene expression, and thus affect risk of DDH, through epigenetic mechanisms, and the phenomenon of breech positioning could be due to genetic risk factors of both mother and child. Such complex genetics together with mechanical factors are consistent with the spectrum of initial presentation of DDH and its later evolvement into secondary OA [39].

In this study, we critically reviewed the current literature on genetics of DDH and OA. While several studies suffer from the lack of power and low sample sizes, several promising initial findings were reported indicating a strong genetic component in this complex disorder. A collective, international effort to increase the sample size is important in the effort to gain knowledge of the genetics of hip dysplasia. Typically, sound genetic studies require thousands of individuals across multiple populations. Currently, the majority of genetic studies on DDH is done in Han Chinese populations. Diversifying the studies across multiple populations would greatly aid our understanding of DDH etiology, also ensuring scientific and health care equality. Larger sample sizes will increase the power to detect true associations with genetic variants of smaller effect size, which is typical of multifactorial, heterogenous disorders such as DDH and OA [40]. As seen in OA, the number of individuals needed to detect robust findings are in the tens of thousands, which indicates the amount of effort needed by the DDH research community [34].

Further, standardizing the study definition of DDH through objective measures, such as radiological measures is also important. Ideally, a measurement representing the three-dimensional structure of both the acetabular and femoral side of the hip joint would be used, incorporating several aspects of joint congruity. Additionally, the genetic studies on DDH and OA would also benefit from good statistical practices, such as adjusting for sex ratios in study groups and correction for multiple testing. Given the known increased prevalence of DDH in females, sex ratio adjustment is important to avoid confounders and spurious associations.

**Fig. 3:**
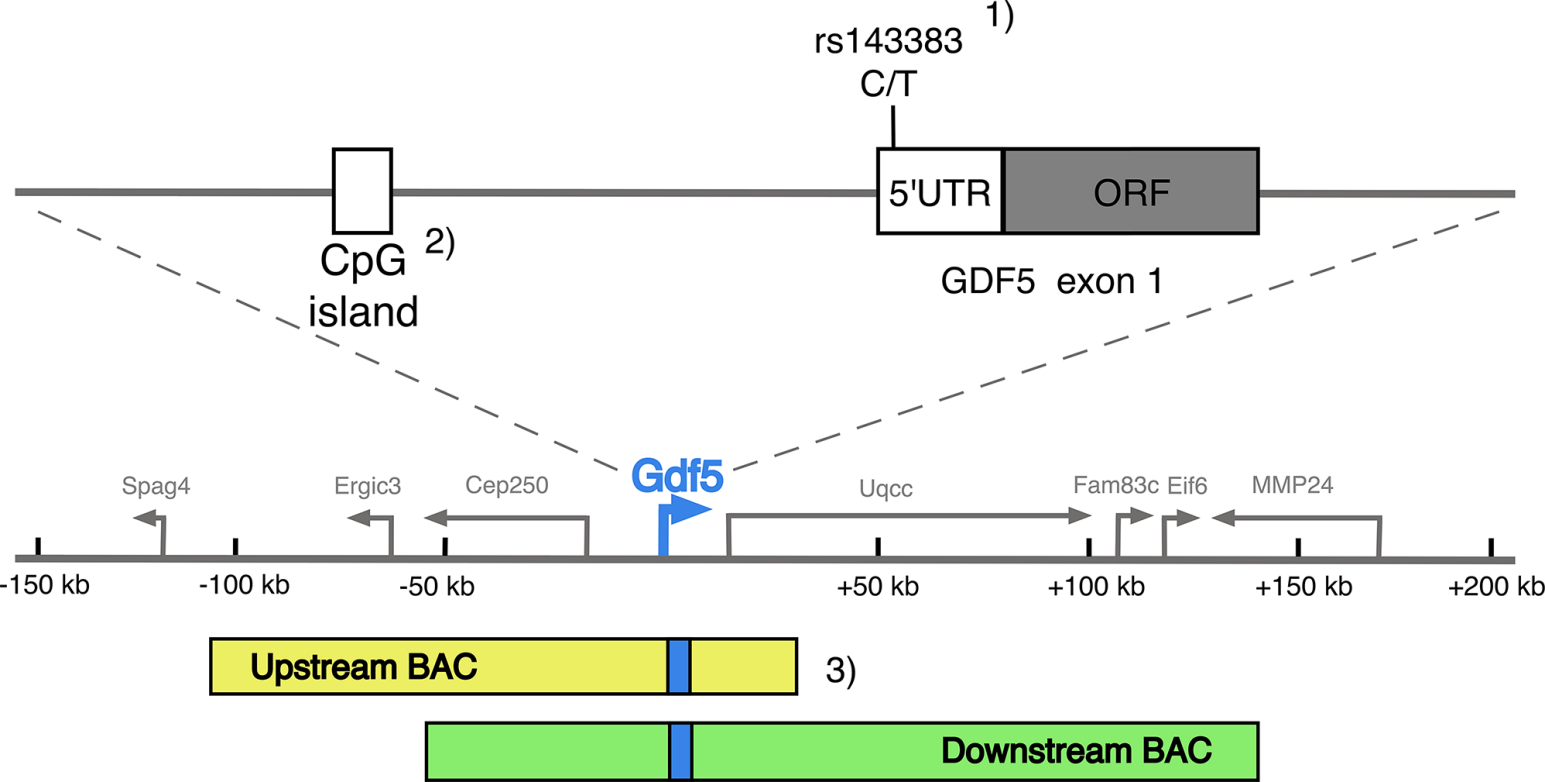
Detailing the relation between GDF5, the SNP rs143383 and regulatory elements including CpG islands. 1) The location of rs143383 within the gene region. The SNP is associated with both DDH, OA and hip shape [15, 16, 51] (2) The SNP is itself a methylation site, and methylation of both this site and the promoter affects GDF5 expression through complex mechanisms elucidated by Reynard et al. in their papers from 2011 and 2014 [44, 45] Baghdadi et al. found that the promoter was hypermethylated in DDH [46] 3) There are several regulatory elements and influences on GDF5 expression, many of which are joint-specific and affect cartilage development and joint shape [21, 41–43]. Figure adapted from figures by Chen and Reynard. DDH: Developmental dysplasia of the hip; OA: Osteoarthritis; SNP: Single Nucleotide Polymorphism

To date, the strongest candidate gene for DDH is *GDF5*, with consistent findings in smaller candidate gene studies, in a large GWAS and in functional studies. A possible biological mechanism through which *GDF5* may affect the development of DDH is the regulation of gene expression. There are multiple regulatory elements both upstream and downstream of the *GDF5* gene, with several functional studies linking those regions to joint and bone formation [41]. Further investigations of the *GDF5* locus revealed a complex relationship between *GDF5* risk alleles for DDH and OA, aforementioned regulatory elements and methylation in the region, with the latter affecting gene expression [21, 42–44]. For instance, several of the SNPs associated with DDH and OA are within known regulatory areas, such as CpG islands, which are DNA regions whose metylation affect gene expression [45]. *GDF5* is differentially expressed in cell cultures depending on the presence of risk alleles and methylation levels in the 5’ untranslated region (UTR) and upstream region of *GDF5*. This occurs in a complex manner that seems to differ between DDH and OA femoral head cartilage [21, 33, 44–46]. Such differential expression could be due to gross morphological differences in the joint shape of individuals with DDH. Increased GDF5 expression is important for cartilage growth and repair and studies have shown decrease in its expression in DDH and OA [21, 41, 42, 47, 48]. Indeed, intraarticular injection of GDF5 protein promotes cartilage repair, one of the avenues that could be taken in the development of therapy and management of DDH and subsequent secondary OA [49, 50]. The expression levels of *GDF5* in the hip joint of mice affect joint morphology, with knock-out of regulatory elements of *GDF5* creating a DDH-like phenotype with a shallow, dysmorphic acetabulum [43]. Figure 3 shows the relationship between SNP association results and functional studies on gene expression in *GDF5.* In summary, aspects of *GDF5* involvement in DDH includes effects on both cartilotype and morphotype, in accordance with Hogervorts theory, where the effects of GDF5 on cartilage could be potential therapeutic target [39, 49, 50].

Importantly, in the Hatzikotoulas study, SNPs in *GDF5* and other associated variants explained only 0.96% of the heritability of DDH, implying a large, undiscovered residual genetic contribution to DDH. This supports the need for a substantial effort in the field of genetics of DDH in order to elucidate the biologic background of DDH. In our future studies, we hope to contribute to this effort and invite fellow researchers to join.

## Electronic supplementary material

Below is the link to the electronic supplementary material.

Supplementary Material 1

## Data Availability

Data sharing is not applicable to this article as no datasets were generated or analyzed during the current study. All data mentioned is available in the corresponding references.

## Abbreviations

AHI: Acetabular Head Index
AI: Acetabular Index
CEA: Center Edge Angle
DDH: Developmental dysplasia of the hip
ECM: Extracellular matrix
THE: Horizontal Toit Extern angle
GWAS: Genome-wide association study
OA: Osteoarthritis
OR: Odds ratio
SNP: Single nucleotide polymorphism
THA: Total hip arthroplasty
UTR: Untranslated region
