# Supplementary material for: Genetics of hip dysplasia – a systematic literature review
Source: BMC Musculoskelet Disord. 2024 Oct 1;25:762. doi: 10.1186/s12891-024-07795-2 (PMC11445845; doi:10.1186/s12891-024-07795-2)
Supplement: Supplementary file 1 — Supplementary Material 1 [file 12891_2024_7795_MOESM1_ESM.docx]

| Supplementary table 1: Detailed overview of the 31 SNP based papers included in the review | | | | | | | | | | | | | | | | |
| --- | --- | --- | --- | --- | --- | --- | --- | --- | --- | --- | --- | --- | --- | --- | --- | --- |
| **#** | **Paper** | **Subanalysis** | **Ethnicity** | **Type of study** | **No of cases** | **% females** | **No of controls** | **% females** | **Gender control** | **Supercontrols (No OA)** | **Multiple testing correction** | **Associated SNP(s)** | **Associated gene(s)** | **Subgroup** | **OR  (95% CI)** | **P-value** |
| 1 | Cengic et al, Int Orthopedics 2015 |  | Slavic | Candidate gene | 68 | 0.22 | 152 | 0.34 | Yes | OA patients only | No | rs1800470 | TGFB1 | CC vs others | 2.42 (1.08-5.43) | 0.032 |
|  | Cengic et al, Int Orthopedics 2015 |  |  |  |  |  |  |  |  |  |  | rs1800796 | IL6 | CC vs others | 6.36 (2.57-15.7) | <0.001 |
| 2 | Dai et al, Arthritis Res Ther. 2008 |  | Han Chinese | Candidate gene | 338 | 0.86 | 622 | 0.51 | Subgroup analyses | No | No | rs143383 | GDF5 | Allelic all | 1.4 (1.11-1.75) | 0.0037 |
|  | Dai et al, Arthritis Res Ther. 2008 |  |  |  |  |  |  |  |  |  |  |  |  | Allelic female | 1.46 (1.21-1.91) | 0.0053 |
|  | Dai et al, Arthritis Res Ther. 2008 |  |  |  |  |  |  |  |  |  |  |  |  | Allelic male | 1.27 (0.75-2.14) | 0.38 |
| 3 | Ghosh et al, Med Sci Monit |  | Western European | Candidate gene | 45 | 0.91 | 95 | 0.52 | No | Yes | No | No association with FBN1 |  |  |  |  |
| 4 | Gumus et al, Indian J Orthop 2021 |  | Turkish | Candidate gene | 68 | 0.48 | 100 | 0.52 | No | Yes | No | No association with BMP2 or UQCC1 |  |  |  |  |
|  | Gumus et al, Indian J Orthop 2021 |  |  |  |  |  |  |  |  |  |  | rs3732378 | CX3CR1 | GG vs others | 75.5 (10-565) | <0.001 |
| 5 | Hao et al, J Orthop Res. 2014 |  | Han Chinese | Candidate gene | 460 | 0.75 | 562 | 0.60 | Subgroup analyses | No | No | rs2303486 | HOXB9 | Genotype all | 1.32 (1.02-1.71) | 0.037 |
|  | Hao et al, J Orthop Res. 2014 |  |  |  |  |  |  |  |  |  |  |  |  | Genotype female | 1.46 (1.08-1.98) | 0.015 |
|  | Hao et al, J Orthop Res. 2014 |  |  |  |  |  |  |  |  |  |  |  |  | Genotype dislocated | 1.35 (1.01-1.80) | 0.042 |
| 6 | Harsanyi et al, Ortop Trauma Rehab 2021 |  | Slavic | Candidate gene | 35 | 0.88 | 83 | 0.6 | No | Yes | No | No association with GDF5 |  |  |  |  |
| 7 | Harsanyi et al, Ortop Trauma Rehab 2021b |  | Slavic | Candidate gene | 45 | 0.84 | 85 | 0.6 | No | Yes | No | No association with IL6 or PAPPA2 |  |  |  |  |
|  | Harsanyi et al, Ortop Trauma Rehab 2021b |  |  |  |  |  |  |  |  |  |  | rs143383 | GDF5 | Genotype all | NA | 0.047 |
| 8 | Hatzikotoulas et al, Commun Biol. 2018 |  | Western European | GWAS | 770 |  | 3364 |  | Yes | Excluding diagnoses | Yes | rs143384 | GDF5 | Allelic all | 1.57 (1.3-1.77) | 1.72E-10 |
|  | Hatzikotoulas et al, Commun Biol. 2018 | Replication | Western European | GWAS | 1129 |  | 4652 |  | Yes | Excluding diagnoses | Yes | rs143384 | GDF5 | Allelic all | 1.37 (1.24-1.51) | 1.33E-10 |
|  | Hatzikotoulas et al, Commun Biol. 2018 | Metaanalysis | Western European | GWAS | 1899 |  | 8016 |  |  |  | Yes | rs143384 | GDF5 | Allelic all | 1.44 (1.34-1.56) | 3.55E-22 |
|  | Hatzikotoulas et al, Commun Biol. 2018 | Metaanalysis | Western European | GWAS |  |  |  |  |  |  |  | rs12479765 | MMP24 | Allelic all | 1.33 (1.20-1.47) | 3.18E-08 |
|  | Hatzikotoulas et al, Commun Biol. 2018 | Metaanalysis | Western European | GWAS |  |  |  |  |  |  |  | rs2050729 | RMB39 | Allelic all | 1.41 (1.25-1.58) | 1.15E-08 |
| 9 | Igrek et al, Acta Chir Orthop Traumatol Chec 2021 |  | Slavic | Candidate gene | 105 | 0.89 | 119 | 0.75 | Subgroup analyses | Yes | No | No association with TGFB1 or IL6 |  |  |  |  |
| 10 | Jawadi et al, J Genet. 2018 |  | Arabic | Candidate gene | 50 | 0.82 | 50 |  | No | No | No | No association with VDR |  |  |  |  |
| 11 | Jia et al, Osteoarthritis Cartilage. 2012 |  | Han Chinese | Candidate gene | 310 | 0.82 | 487 | 0.53 | Yes | No | No | rs726252 | PAPPA2 | Allelic all | 1.83 (1.33-2.52) | 0.001 |
|  | Jia et al, Osteoarthritis Cartilage. 2012 |  |  |  |  |  |  |  |  |  |  |  |  | Allelic female | 1.605 (1.33-2.28) | 0.008 |
|  | Jia et al, Osteoarthritis Cartilage. 2012 |  |  |  |  |  |  |  |  |  |  |  |  | Allelic male | 3.69 (1.45-9.38) | 0.006 |
| 12 | Kapoor et al, J Negat Results Biomed. 2007 |  | Western European | Candidate gene | 45 | 0.91 | 101 | 0.53 | No? | Yes | Yes | No association with ER or VDR |  |  |  |  |
| 13 | Kolundžić et al, Cytokine. 2011 |  | Slavic | Candidate gene | 28 | 0.82 | 20 | 0.55 | Yes | Yes | No | rs1800470 | TGFB1 | Allelic all | 13.4 (1.6-110) | 0.016 |
|  | Kolundžić et al, Cytokine. 2011 |  |  |  |  |  |  |  |  |  |  | rs1800796 | IL6 | Allelic all | 6.2 (1.3-30.0) | 0.024 |
| 14 | Li et al, J Orthop Res. 2017 |  | Han Chinese | Candidate gene | 689 | 0.82 | 689 | 0.44 | Yes | Yes | No | rs3732378 | CX3CR1 | Allelic all | 2.25 (1.42-3.56) | 0.001 |
|  | Li et al, J Orthop Res. 2017 |  |  |  |  |  |  |  |  |  |  | rs3732379 | CX3CR1 | Allelic all | 1.84 (1.19-2.84) | 0.006 |
| 15 | Ma et al, Sci Rep. 2017 |  | Han chinese | Candidate gene | 373 | 0.79 | 1115 | 0.79 | Yes | No | Yes | rs1800470 | TGFB1 | Allelic all | 1.37 (1.12-1.68) | 0.002 |
|  | Ma et al, Sci Rep. 2017 | Replication, results from combined sample | Han chinese |  | 691 | 0.84 | 2027 | 0.84 | Yes | No | Yes | rs1800470 | TGFB1 | Allelic all | 1.26 (1.11-1.42) | 0.0004 |
| 16 | Qiao et al, Int J Exp Pathol 2017a | Sample A | Han Chinese | Candidate gene | 409 | 0.90 | 351 | 0.38 | Subgroup analyses | No | No | No association with CX3CR1 |  |  |  |  |
| 17 | Qiao et al, Int J Exp Pathol 2017b | Sample A | Han Chinese | Candidate gene | 386 | ? | 558 | ? | Subgroup analyses | No | No |  |  |  |  |  |
|  | Qiao et al, Int J Exp Pathol 2017b | Sample B | Han Chinese | Candidate gene | 599 | ? | 1485 | ? | Subgroup analyses | No | No |  |  |  |  |  |
|  | Qiao et al, Int J Exp Pathol 2017b | Total sample | Han Chinese | Candidate gene | 984 | 0.87 | 2043 | 0.42 | Subgroup analyses | No | No | rs10250905 | TXNDC3 | Allelic all | 0.79 (0.62-0.93) | 1.53E-05 |
| 18 | Rouault et al, Osteoarthritis Cartilage 2009 |  | French | Candidate gene | 239 | 0.91 | 239 | 0.91 | Yes | Yes | Yes | No association with HOXB9 |  |  |  |  |
|  | Rouault et al, Osteoarthritis Cartilage 2009 |  |  |  |  |  |  |  |  |  |  | No association with COL1A1 |  |  |  |  |
| 19 | Rouault et al, Osteoarthritis Cartilage 2010 |  | French | Candidate gene | 239 | 0.91 | 239 | 0.91 | Yes | Yes | Yes | rs143384 | GDF5 | Allelic all | 1.53 (1.18-1.98) | 0.0020 |
| 20 | Sadat-Ali et al, Biocehm genet 2018 |  | Arabic | Candidate gene | 100 |  | 100 |  | No | ? | No | No association with GDF5 |  |  |  |  |
| 21 | Shi et al, BioMed Res Int 2014 |  | Han Chinese | Candidate gene | 697 | 0.85 | 707 | 0.42 | Subgroup analyses | No | No | No association with PAPPA2 |  |  |  |  |
| 22 | Sun et al, PLoS ONE 2015 |  | Han Chinese | GWAS | 386 | ? | 558 | ? | No | No | Partially | No genome-wide significant hits |  |  |  |  |
|  | Sun et al, PLoS ONE 2015 | Replication, results from combined sample | Han Chinese |  | 755 | ? | 944 | ? | No | No | No | rs6060373 | UQCC | Allelic all | 1.35 (1.19-1.53) | 3.63E-06 |
| 23 | Sun et al, Sci Rep 2019 |  | Han Chinese | Candidate gene | 386 | ? | 558 | ? | No | No | No | rs3782499 | WIF1 | Allelic all | 0.68 (0.53-0.87) | 0.002 |
|  | Sun et al, Sci Rep 2019 | Replication, results from combined sample | Han Chinese |  | 200 | ? | 429 | ? | No | No | No | rs3782500 | WIF2 | Allelic all | 0.67 (0.55-0.81) | 4.37E-05 |
| 24 | Tian et al, BMC Musculoskelet Disord 2012 |  | Han Chinese | Candidate gene | 209 | 1.00 | 173 | 1.00 | Yes | No | No | No association with HOXD9 |  |  |  |  |
| 25 | Wang K et al, Osteoarthritis Cartilage 2010 |  | Han Chinese | Candidate gene | 505 | 0.86 | 551 | ? | Yes | No | No | No association with TBX4 |  |  |  |  |
| 26 | Xu et al, Int J Exp Pathol 2016 |  | Han Chinese | Candidate gene | 170 | 0.90 | 454 | 0.32 | Subgroup analyses | No | No | No association with SMOC1 |  |  |  |  |
| 27 | Xu et al, Aging, 2020 |  | Han Chinese | Candidate gene | 350 | 0.89 | 595 | 0.75 | Subgroup analyses | No | No | rs927793 | COL11A2 | Genotype female | NA | 0.006 |
| 28 | Yan et al, Clinical Genet 2019 |  | Han Chinese | GWAS | 386 | ? | 500 | ? | Yes | No | Partially | rs61930502 | RASAL1, SDSL | Allelic all | 1.8 | 2.65E-07 |
|  | Yan et al, Clinical Genet 2019 |  | Han Chinese | GWAS | 574 | ? | 569 | ? | Yes | No | Partially | rs61930502 | RASAL1, SDSL | Allelic all | 0.718 | 1.86E-04 |
| 29 | Zhang et al, Gene 2018 |  | Han Chinese | Candidate gene | 386 |  | 558 |  | No | No | No | rs1230345 | WISP3 | Allelic all | 0.71 (0.58-0.87) | 0.000707 |
| 30 | Zhao et al, Sci China Life Sci 2013 |  | Han Chinese | Candidate gene | 192 | 1.00 | 191 | 1.00 | Yes | No | Yes | rs224332 | GDF5 | Allelic all | NA | 0.001 |
|  | Zhao et al, Sci China Life Sci 2013 |  |  |  |  |  |  |  |  |  |  | rs224333 | GDF5 | Allelic all | NA | 0.006 |
| 31 | Zhu et al, Rheumatol Int 2011 |  | Han Chinese | Candidate gene | 368 | 0.85 | 413 | 0.74 | Subgroup analyses | No | No | No association with DVWA |  |  |  |  |

SNP: Single Nucleotide Polymorphism, OR: Odds Ratio, OA: Osteoarthritis, GWAS: Genome Wide Association Study
